# Factors Associated With Urgent Care Reliance and Outpatient Health Care Use Among Children Enrolled in Medicaid

**DOI:** 10.1001/jamanetworkopen.2020.4185

**Published:** 2020-05-06

**Authors:** Rebecca R. Burns, Elizabeth R. Alpern, Jonathan Rodean, Therese Canares, Brian R. Lee, Matt Hall, Amanda Montalbano

**Affiliations:** 1 Division of Emergency Medicine, Ann & Robert H. Lurie Children’s Hospital, Chicago, Illinois; 2 Division of Analytics, Children’s Hospital Association, Lenexa, Kansas; 3 Division of Emergency Medicine, Johns Hopkins University School of Medicine, Baltimore, Maryland; 4 Health Outcomes and Health Services Research, Children’s Mercy, Kansas City, Missouri; 5 Division of Urgent Care, Children’s Mercy, Kansas City, Missouri

## Abstract

**Importance:**

Urgent care (UC) centers are a growing option to address children’s acute care needs, which may cause unanticipated changes in health care use.

**Objectives:**

To identify factors associated with high UC reliance among children enrolled in Medicaid and examine the association between UC reliance and outpatient health care use.

**Design, Setting, and Participants:**

A retrospective cohort study used deidentified data on 4 133 238 children from the Marketscan Medicaid multistate claims database to calculate UC reliance and outpatient health care use. Children were younger than 19 years, with 11 months or more of continuous Medicaid enrollment and 1 or more UC, emergency department (ED), primary care provider (PCP; physician, advanced practice nurse, or physician assistant; well-child care [WCC] or non-WCC), or specialist outpatient visit during the 2017 calendar year. Statistical analysis was conducted from November 11 to 26, 2019.

**Exposures:**

Urgent care, ED, PCP (WCC and non-WCC), and specialist visits based on coded location of services.

**Main Outcomes and Measures:**

Urgent care reliance, calculated by the number of UC visits divided by the sum of total outpatient (UC, ED, PCP, and specialist) visits. High UC reliance was defined as UC visits totaling more than 33% of all outpatient visits.

**Results:**

Of 4 133 238 children in the study, 2 090 278 (50.6%) were male, with a median age of 9 years (interquartile range, 4-13 years). A total of 223 239 children (5.4%) had high UC reliance. Children 6 to 12 years of age were more likely to have high UC reliance compared with children 13 to 18 years of age (adjusted odds ratio, 1.07; 95% CI, 1.06-1.09). Compared with white children, black children (adjusted odds ratio, 0.81; 95% CI, 0.81-0.82) and Hispanic children (adjusted odds ratio, 0.61; 95% CI, 0.60-0.61) were less likely to have high UC reliance. Adjusted for age, sex, race/ethnicity, and presence of chronic or complex conditions, children with high UC reliance had significantly fewer PCP encounters (WCC: adjusted rate ratio, 0.60; 95% CI, 0.60-0.61; and non-WCC: adjusted rate ratio, 0.41; 95% CI, 0.41-0.41), specialist encounters (adjusted rate ratio, 0.31; 95% CI, 0.31-0.31), and ED encounters (adjusted rate ratio, 0.68; 95% CI, 0.67-0.68) than children with low UC reliance.

**Conclusions and Relevance:**

High UC reliance occurred more often in healthy, nonminority, school-aged children and was associated with lower health care use across other outpatient settings. There may be an opportunity in certain populations to ensure that UC reliance does not disrupt the medical home model.

## Key Points

**Question:**

Is high reliance on urgent care facilities by children associated with decreased use of other sites of outpatient health care?

**Findings:**

In a cohort study of 4 133 238 children enrolled in Medicaid, 5% had high reliance on urgent care (defined as >33% of all outpatient visits). High reliance on urgent care was seen more often in healthy, nonminority, school-aged children and was associated with lower health care use across other outpatient settings.

**Meaning:**

Although urgent care facilities may serve to increase access for acute care needs, in certain populations high reliance on urgent care was associated with lower use of other outpatient care sites.

## Teaser

This cohort study examines factors associated with high usual care reliance among children enrolled in Medicaid and examines the association between usual care reliance and outpatient health care use.

## Introduction

With increasing availability and ease of access, urgent care (UC) sites are a growing option for patients to address acute health care needs, including low-acuity illnesses or injuries.^1^ As UC centers become a popular setting for acute care, there is a concern that this convenience may affect the patient’s established relationship with their primary care provider (PCP; physician, advanced practice nurse, or physician assistant). If patients rely on UC for most of their health care needs, it may disrupt the continuity provided in the medical home model and lead to unanticipated changes in health.^2,3^ The association of UC visits with patients’ relationships with their PCP is especially pertinent in pediatrics, where routine well-child care (WCC) visits are paramount to ensuring children’s optimal growth and development. The American Academy of Pediatrics recommends multiple WCC visits per year for children younger than 3 years and yearly WCC visits for those 3 to 21 years of age.^4^ Children regularly seeking acute care outside the PCP’s office may represent missed opportunities for preventive services or identification and management of chronic conditions, which in turn risks fragmenting continuity of care.^2,4^

Previous studies exploring fragmentation of continuity of care evaluated the burden and risk factors of children who frequent the emergency department (ED) but did not evaluate children who frequently use UC.^3,5,6,7,8,9,10^ The literature on health care use makes an important distinction between those who frequently use the ED (high use) and those who rely on the ED for most of their health care needs (high reliance).^10^ High ED reliance describes the proportion of ED visits in relation to all outpatient health care services and can be quantified as the percentage of all ambulatory services that occur in the ED.^10^

The aims of this study were to identify factors associated with high UC reliance and to examine the association between high UC reliance and the use of other sites of outpatient health care. We hypothesized that high UC reliance may be associated with a disruption of the medical home model.

## Methods

### Population and Data Source

We conducted a retrospective cohort study of all children younger than 19 years in the 2017 Marketscan Medicaid multistate claims database (Truven Health Analytics). We included children who had continuous enrollment (defined as ≥11 months of coverage) and had at least 1 ED, UC, PCP, or specialist visit during the 2017 calendar year. In 2017, the Marketscan database contained all inpatient, outpatient, and retail pharmacy claims from 9 deidentified states, as well as enrollment information such as year of birth, sex, race/ethnicity, and the months of enrollment.^11^ The study protocol was reviewed by the Children’s Mercy Kansas City’s institutional review board and deemed nonhuman participants research. This study adhered to the Strengthening the Reporting of Observational Studies in Epidemiology (STROBE) reporting guideline.

### Outcome Measure

The main outcome was UC reliance, adapted from the work of Kroner et al^10^ on ED reliance, defined as the number of UC visits divided by the sum of UC, ED, PCP, and specialist visits. For each child, UC reliance could range from 0% (no reliance; no UC visits regardless of the number of ED, PCP, or specialty visits) to 100% (complete reliance; at least 1 UC visit and no ED, PCP, or specialty visits). Likewise, our definition of high UC reliance was also adapted from this prior work as well as work that a priori determined high ED reliance among young children based on an expert panel of pediatricians as reliance greater than 33%.^10,12^ Reliance on other sources of care (eg, PCP reliance) was calculated similarly.

### Covariates

We examined high and low UC reliance across demographic and clinical characteristics including age, sex, race/ethnicity, presence of a complex chronic condition (CCC), and number of chronic conditions. Children with CCCs^13^ were identified using all diagnoses from all Medicaid claims in the study period. In addition, chronic conditions were identified using the Agency for Healthcare Research and Quality’s Chronic Condition Indicator (CCI),^14^ also using all diagnoses from all claims in the study period. To acknowledge the intersection of the CCC (CCCs such as muscular dystrophy) and CCI categorization (potential non-CCCs such as asthma or allergic rhinitis), as well as the distinct information gained from each, we created a chronic condition profile categorizing into groups children with a CCC and for those without a CCC, the number of CCI conditions (0, 1, 2, or ≥3).^15^

The setting in which the care was delivered (ED, UC, PCP, specialist, or inpatient) was classified based on the coded location of services provided in the claims data.^16^ Primary care provider visits were delineated as WCC and non-WCC visits. Well-child care visits were identified based on recorded *International Classification of Diseases, Ninth Revision* diagnosis codes (V20.2, V20.3, V70.0, V70.3, V70.5, V70.6, V70.8, and V70.9) or *Current Procedural Terminology* codes (99381, 99382, 99383, 99384, 99385, 99391, 99392, 99393, 99394, 99395, 99432, and 99461).^17^

### Statistical Analysis

Statistical analysis was conducted from November 11 to 26, 2019. Descriptive statistics were calculated for the entire study population and stratified based on UC reliance category (low reliance [≤33%] or high reliance [>33%]). Differences in proportions were determined using χ^2^ tests. Multivariable logistic models were then used to provide estimated adjusted odds of high UC reliance. Generalized linear models with an assumption of an underlying Poisson distribution were used to calculate adjusted use rates. Age, sex, race/ethnicity, chronic condition profile, and hospitalization in the study period were covariates in the models. We performed sensitivity analysis in the generalized linear models, removing ED visits from the determination of UC reliance to validate the association that UC visits had with nonemergency outpatient visits (PCP and specialist). All analyses were performed with SAS 9.4 (SAS Institute Inc). *P* values were from 2-sided tests and results were deemed statistically significant at *P* < .05.

## Results

During the study period, 6 596 754 enrollees younger than 19 years were identified in the database, approximately 14% of the total number of children with public health coverage nationally for the year 2017.^18^ Of these, 4 133 238 children (62.7%) met inclusion criteria; 2 090 278 children (50.6%) were male and the median age was 9 years (interquartile range, 4-13 years) (Table 1). In this study cohort, 3 618 527 children (87.5%) had no UC visits, 329 734 (8.0%) had 1 UC visit, 106 175 (2.6%) had 2 UC visits, and 78 802 (1.9%) had 3 or more UC visits. The high UC reliance group (n = 223 239) accounted for 5.4% of the total study population. Demographic and clinical characteristics of the overall, low UC reliance, and high UC reliance groups are presented in Table 1.

**Table 1.** Characteristics of Study Population Associated With Urgent Care Reliance

Characteristic	Children, No. (%)		
	Overall (N = 4 133 238)	Urgent care reliance	
		Low (n = 3 909 999 [94.6%])	High (n = 223 239 [5.4%])
Age, y^a^			
<1	46 168 (1.1)	45 883 (1.2)	285 (0.1)
1-2	539 702 (13.1)	523 378 (13.4)	16 324 (7.3)
3-5	753 436 (18.2)	711 137 (18.2)	42 299 (18.9)
6-12	1 648 747 (39.9)	1 547 315 (39.6)	101 432 (45.4)
13-18	1 145 185 (27.7)	1 082 286 (27.7)	62 899 (28.2)
Sex^a^			
Male	2 090 278 (50.6)	1 978 851 (50.6)	111 427 (49.9)
Female	2 042 960 (49.4)	1 931 148 (49.4)	111 812 (50.1)
Race/ethnicity^a^			
White	1 855 475 (44.9)	1 740 290 (44.5)	115 185 (51.6)
Black	1 295 053 (31.3)	1 232 751 (31.5)	62 302 (27.9)
Hispanic	349 428 (8.5)	366 695 (8.6)	12 733 (5.7)
Other	144 349 (3.5)	137 578 (3.5)	6771 (3.0)
Missing	488 933 (11.8)	462 685 (11.8)	26 248 (11.8)
Any CCC^a^			
No	3 916 570 (94.8)	3 697 284 (94.6)	219 286 (98.2)
Yes	216 668 (5.2)	212 715 (5.4)	3953 (1.8)
No. of CCIs^a^			
0	1 822 999 (44.1)	1 694 322 (43.3)	128 677 (57.6)
1	1 230 410 (29.8)	1 169 693 (29.9)	60 717 (27.2)
2	566 549 (13.7)	545 116 (13.9)	21 433 (9.6)
≥3	513 280 (12.4)	500 868 (12.8)	12 412 (5.6)

Abbreviations: CCC, complex chronic condition; CCI, chronic condition indicator.

^a^ All comparisons between low and high reliance categories were significant at *P* < .001.

In multivariable analysis, high UC reliance was associated with age, race/ethnicity, and presence of any CCI condition or CCC (Table 2). Grade school–aged children (age, 6-12 years) were more likely to have high UC reliance compared with children aged 13 to 18 years (adjusted odds ratio [aOR], 1.07; 95% CI, 1.06-1.09). Compared with white children, children who were black (aOR, 0.81; 95% CI, 0.81-0.82) or of Hispanic ethnicity (aOR, 0.61; 95% CI, 0.60-0.61) were less likely to have high UC reliance. Children with any CCI condition or CCC were also less likely to have high UC reliance. We performed a sensitivity analysis by removing ED visits from the equation (eTable in the Supplement). An additional 37 391 enrollees were in the high UC reliance group, but there were no substantive changes in the model.

**Table 2.** Multivariable Analysis of Factors Associated With High Urgent Care Reliance

Characteristic	High urgent care reliance, aOR (95% CI)
Age, y	
<1	0.11 (0.10-0.12)
1-2	0.41 (0.40-0.42)
3-5	0.86 (0.85-0.87)
6-12	1.07 (1.06-1.09)
13-18	1 [Reference]
Sex	
Male	0.97 (0.97-0.98)
Female	1 [Reference]
Race/ethnicity	
White	1 [Reference]
Black	0.81 (0.81-0.82)
Hispanic	0.61 (0.60-0.61)
Other	0.77 (0.75-0.79)
Missing	1.07 (1.06-1.09)
Chronic condition profile	
No CCCs	
0 Chronic conditions	1 [Reference]
1 Chronic condition	0.61 (0.60-0.61)
2 Chronic conditions	0.42 (0.43-0.43)
≥3 Chronic conditions	0.29 (0.28-0.29)
With a CCC	0.21 (0.20-0.21)

Abbreviations: aOR, adjusted odds ratio; CCC, complex chronic condition.

**Figure 1.**
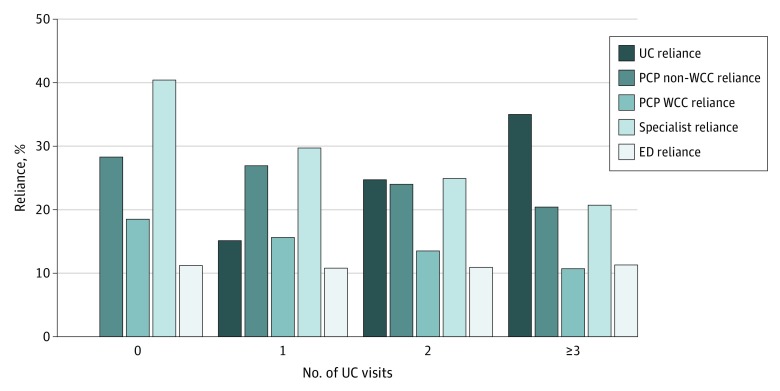
Health Care Site Reliance and Urgent Care (UC) Use Among 4 133 238 Medicaid Recipients Younger Than 19 Years, 2017 ED indicates emergency department; PCP, primary care provider (physician, advanced practice nurse, or physician assistant); and WCC, well-child care.

**Figure 2.**
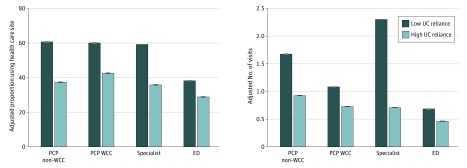
Health Care Site Use Associated With Urgent Care (UC) Reliance Among 4 133 238 Medicaid Recipients Younger Than 19 Years, 2017 ED indicates emergency department; PCP, primary care provider (physician, advanced practice nurse, or physician assistant); and WCC, well-child care. Error bars indicate 95% CIs.

In addition, with increased UC use, children had differing health care setting reliance. Primary care provider WCC, PCP non-WCC, and specialist reliance all declined with increasing UC use (Figure 1). After adjusting for enrollee characteristics, children with high UC reliance had significantly lower use than children with low UC reliance of all other sources of outpatient care, both in the proportion accessing care at these sites (PCP WCC: aOR, 0.48; 95% CI, 0.48-0.49; PCP non-WCC: aOR, 0.38; 95% CI, 0.37-0.38; specialist: aOR, 0.36; 95% CI, 0.36-0.36; and ED: aOR, 0.66; 95% CI, 0.65-0.66) and in the number of visits (PCP WCC: adjusted rate ratio [aRR], 0.60; 95% CI, 0.60-0.61; PCP non-WCC: aRR, 0.41; 95% CI, 0.41-0.41; specialist: aRR, 0.31; 95% CI, 0.31-0.31; and ED: aRR, 0.68; 95% CI, 0.67-0.68) (Figure 2). Although ED use remained steady across increasing UC use (Figure 1), children in the high UC reliance group had a lower proportion using the ED and fewer ED visits compared with children in the low UC reliance group (Figure 2).

## Discussion

Our study found that high UC reliance was associated with lower use of all other sites of outpatient care, including the PCP and ED. We also identified that increasing UC use was associated with declining reliance on other sources of care. Although high UC reliance represented a small percentage of the study population, its association was seen across the entire medical neighborhood. The number of UC centers increased from 6946 in 2015 to 9272 in 2019.^19^ Urgent care sites as a setting for acute care are experiencing rapid growth, which may serve to magnify these findings.^20^

Past work indicates that high UC use is associated with high use of all outpatient health care sites, including the PCP and ED^15^; however, we found that those who rely on UC for more than 33% of their acute care needs have fewer visits to the PCP and ED. Urgent care sites may be filling a gap in access to care for certain populations.^15,21^ We found that high UC reliance in the Medicaid population occurs more often in healthy, school-aged children. The potentially most vulnerable patients—younger children, minority groups, and those with 3 or more CCIs or a CCC—had the least reliance on UC. Our findings on UC reliance were similar to findings by Kroner et al^10^ on pediatric ED reliance, which indicated that younger children and those with a CCC have lower ED reliance. However, in that study, black children were found to have higher ED reliance,^10^ which is different from our findings on UC reliance.^22,23^ Additional investigation into the location of UC sites relative to minority populations may clarify the reasons for this difference.^24^

Further study of factors associated with high UC reliance is needed to assess if and how reliance on UC may be associated with a child’s relationship with the medical home. The data set used for this study does not capture nonbillable communication with the PCP, who may be counseling and coordinating where patients are seeking health care. A recent American Academy of Pediatrics policy statement endorses the “medical home as the best location for children to receive care for an acute nonemergent health concern” to achieve the “optimal clinical and long term health outcomes.”^25^ However, patients frequently seek care outside the traditional hours of operation of a primary care office, when they may receive acute care treatment at non-PCP sites. We found that patients who are younger or have complex medical problems rely more heavily on their medical home, regardless of whether they see a generalist or specialist, which may indicate that these patients are in closer communication with their PCPs.

Because UC is a relatively new site of care, there are no studies in the literature, to our knowledge, to help determine what constitutes a high level of reliance on UC. Because the ED is the historic site of episodic acute care, we based our definitions of high and low reliance on similar previous ED investigations with the definition of high UC reliance set at more than 33% for all ages.^10,12^ However, older children have fewer recommended WCC visits with their PCP and many do not complete annual PCP visits. For fiscal year 2013, 60% of children covered by Medicaid had at least 6 WCC visits in the first 15 months of life, but less than half of adolescents had at least 1 WCC visit.^26^ As a result, older children may fall into the high UC reliance group with fewer UC visits owing to fewer potential PCP visits in the denominator. Future evaluation of a higher threshold constituting high UC reliance for older children should be explored.

Use of the Medicaid database permitted us to study a large population and differentiate UC visits from visits to other acute care locations. Furthermore, the population insured by Medicaid has been previously identified to seek care outside the medical home, and to be at risk of becoming reliant on acute care sites such as the ED or UC; thus, this population warranted investigation in our study.^10,27^

### Limitations

There are several limitations that should be considered when interpreting these results. The type of UC center (independent or associated with the health care system of the patient’s PCP) is not distinguished in the data set. Urgent care centers existing within a particular health care system may allow UC clinicians to access the patient’s medical record and facilitate communication with the PCP. Also, the database does not differentiate whether a patient had Medicaid managed care or Medicaid fee for service; therefore, we were unable to determine whether type of Medicaid coverage was associated with reliance. In addition, Medicaid makes up a small portion of overall UC visits, and analyzing data from a subset of a single public insurer may affect the generalizability of the results.^28^ The data represent a 1-year period, which may not be long enough to fully account for patients’ ongoing outpatient health care use patterns. Also, the inclusion criteria of 11 months of continuous enrollment in Medicaid limited our sample of infants. Finally, as the focus of our study was on patients seeking outpatient care, we can comment only on children with at least 1 outpatient visit. Reliance would be incalculable without any outpatient visits, as the denominator would be zero.^10^

## Conclusions

High UC reliance was associated with lower health care use across other outpatient care sites, including PCP and ED visits. High UC reliance was relatively uncommon in the Medicaid population but more common in healthy, nonminority, school-aged children. High UC reliance likely fills a need for children with acute care issues but has the potential to disrupt the medical home model. Further studies are needed to investigate the reasons that patients and families seek care at UC sites and evaluate the health and financial implications of this choice.

## Notes

Supplement. **eTable.** Sensitivity Multivariable Analysis of Risk Factors of High Urgent Care Reliance Without ED Visits in the Model
