# Supplementary material for: Factors Associated With Urgent Care Reliance and Outpatient Health Care Use Among Children Enrolled in Medicaid
Source: JAMA Netw Open. 2020 May 6;3(5):e204185. doi: 10.1001/jamanetworkopen.2020.4185 (PMC7203605; doi:10.1001/jamanetworkopen.2020.4185)
Supplement: Supplement. — eTable. Sensitivity Multivariable Analysis of Risk Factors of High Urgent Care Reliance Without ED Visits in the Model [file jamanetwopen-3-e204185-s001.pdf]

Supplementary Online Content

Burns RR, Alpern ER, Rodean J, et al. Factors associated with urgent care reliance and outpatient health care use among children enrolled in Medicaid. *JAMA Netw Open.* 2020;3(5):e204185. doi:10.1001/jamanetworkopen.2020.4185

eTable. Sensitivity Multivariable Analysis of Risk Factors of High Urgent Care Reliance Without ED Visits in the Model

This supplementary material has been provided by the authors to give readers additional information about their work.

© 2020 Burns RR et al. *JAMA Network Open.*

eTable. Sensitivity Multivariable Analysis of Risk Factors of High Urgent Care Reliance Without ED Visits in the Model

	**High UC Reliance (>33%)**	
	**aOR**	**95% CI**
Age, years		
<1	0.13	(0.12, 0.15)
1-2	0.46	(0.45, 0.47)
3-5	0.88	(0.87, 0.89)
6-12	1.03	(1.02, 1.04)
13-18	Ref	
Gender		
Male	0.98	(0.97, 0.99)
Female	Ref	
Race/Ethnicity		
White	Ref	
Black	0.86	(0.86, 0.87)
Hispanic	0.59	(0.58, 0.60)
Other	0.75	(0.73, 0.76)
Missing	1.12	(1.10, 1.13)
Chronic Condition Profile		
No CCC, 0 Chronic Conditions	Ref	
No CCC, 1 Chronic Condition	0.61	(0.60, 0.61)
No CCC, 2 Chronic Conditions	0.45	(0.44, 0.45)
No CCC, 3+ Chronic Conditions	0.32	(0.32, 0.33)
With a CCC	0.23	(0.23, 0.24)
Any Hospitalization in Study Period		
No	Ref	
Yes	0.93	(0.90, 0.96)

aOR, adjusted odds ratio; CCC, complex chronic conditions; CI, confidence interval; UC, urgent care.

© 2020 Burns RR et al. *JAMA Network Open.*
